# Exploring Low-Power Single-Pulsed Laser-Triggered Two-Photon Photodynamic/Photothermal Combination Therapy Using a Gold Nanostar/Graphene Quantum Dot Nanohybrid

**DOI:** 10.1021/acsami.3c03578

**Published:** 2023-04-21

**Authors:** Amir Soleimany, Sepideh Khoee, Sofia Dias, Bruno Sarmento

**Affiliations:** † i3S, Instituto de Investigação e Inovação em Saúde, Universidade do Porto, Rua Alfredo Allen 208, 4200-135 Porto, Portugal; ‡ INEB, Instituto de Engenharia Biomédica, Universidade do Porto, Rua Alfredo Allen 208, 4200-135 Porto, Portugal; § Polymer Laboratory, School of Chemistry, College of Science, University of Tehran, Tehran 14155-6455, Iran; ∥ ICBAS − Instituto de Ciências Biomédicas Abel Salazar, Universidade do Porto, Rua Jorge de Viterbo Ferreira 228, 4050-313 Porto, Portugal; ⊥ IUCS-CESPU, Rua Central de Gandra 1317, 4585-116 Gandra, Portugal

**Keywords:** hyperthermia, phototherapy, plasmonic metal, quantum dots, singlet oxygen, thiolated chitosan

## Abstract

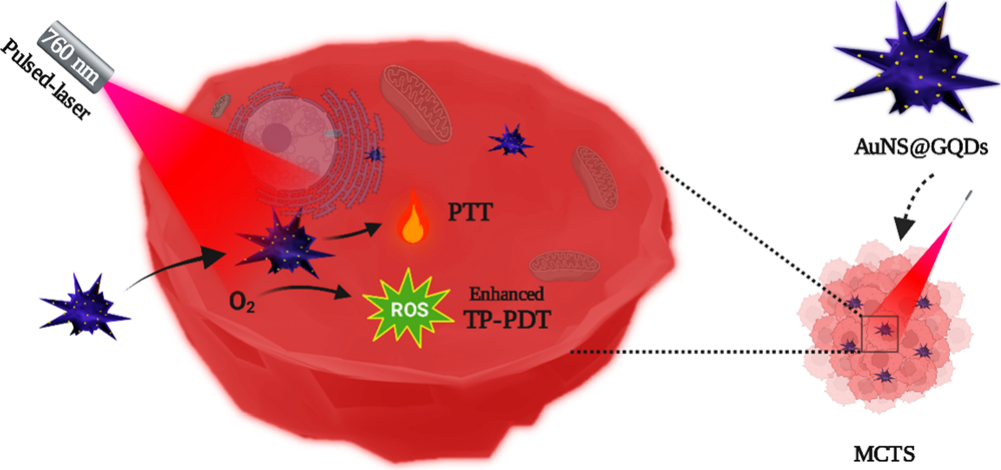

Combined photodynamic/photothermal therapy (PDT/PTT) has emerged as a promising cancer treatment modality due to its potential synergistic effects and identical treatment procedures. However, its clinical application is hindered by long treatment times and complicated treatment operations when separate illumination sources are required. Here, we present the development of a new nanohybrid comprising thiolated chitosan-coated gold nanostars (AuNS-TCS) as the photothermal agent and riboflavin-conjugated N,S-doped graphene quantum dot (Rf-N,S-GQD) as the two-photon photosensitizer (TP-PS). The nanohybrid demonstrated combined TP-PDT/PTT when a low-power, single-pulsed laser irradiation was applied, and the localized surface plasmon resonance of AuNS was in resonance with the TP-absorption wavelength of Rf-N,S-GQD. The TCS coating significantly enhanced the colloidal stability of AuNSs while providing a suitable substrate to electrostatically anchor negatively charged Rf-N,S-GQDs. The plasmon-enhanced singlet oxygen (^1^O_2_) generation effect led to boosted ^1^O_2_ production both extracellularly and intracellularly. Notably, the combined TP-PDT/PTT exhibited significantly improved phototherapeutic outcomes compared to individual strategies against 2D monolayer cells and 3D multicellular tumor spheroids. Overall, this study reveals a successful single-laser-triggered, synergistic combined TP-PDT/PTT based on a plasmonic metal/QD hybrid, with potential for future investigation in clinical settings.

## 1 Introduction

Phototherapy, which includes photothermal therapy (PTT) and photodynamic therapy (PDT), has emerged as a promising treatment modality due to its noninvasiveness, spatiotemporal selectivity, and controllability when compared to traditional therapeutic strategies, including surgery, chemotherapy, and radiotherapy.^1−4^ During PDT, light-excited phototherapeutic agents, also known as photosensitizers (PSs), can transfer energy to surrounding molecular oxygen to primarily generate singlet oxygen (^1^O_2_),^5^ resulting in irreversible tumor ablation via oxidative stress.^6^ Therefore, the effectiveness of PDT depends largely on the availability of oxygen in the tumor. In contrast, PTT is an oxygen-independent therapeutic strategy based on photothermal conversion, where a photothermal agent (PTA) converts the absorbed light into heat, directly causing tumor ablation.^7,8^ Despite significant development, complete treatment of solid tumors through either PDT or PTT can be challenging due to several restrictions, including hypoxia and heat shock proteins (HSP), respectively.^9−11^

Interestingly, PDT/PTT combination therapy has the potential for mutual efficiency enhancement and synergistic therapeutic outcomes that could alleviate the inherent restrictions of each individual strategy.^12^ During PTT, local heat generated in the tumor can improve the intracellular accumulation of the therapeutic agent by increasing the cell membrane permeability and relieving the tumor hypoxic environment by accelerating blood flow in the irradiated region, resulting in enhanced PDT.^13,14^ The HSP protective effect during PTT can also be nullified by the reactive oxygen species (ROS) generated from PDT.^15^ However, to avoid complicated and lengthy treatment procedures of separate activation of PDT and PTT with two different lasers, designing and developing single laser-activatable PTA/PS hybrids is highly desirable.^16,17^

Two-photon mediated (TP-) PDT shows great promise in extending the excitation wavelength of PSs into the biological window, which overcomes the limited penetration depth of UV–vis light.^18,19^ TP excitation (TPE) is a nonlinear optical phenomenon that involves the absorption of two lower-energy photons simultaneously, as opposed to the usual one-photon excitation (OPE).^20^ Despite the advantages over one-photon-PDT, the clinical application of TP-PDT is impeded by the low two-photon absorption cross-section (TPAC) of the conventional PSs.^21^ To bypass the low TPAC of PSs, indirect excitation through fluorescence (FL) resonance energy transfer is proposed when they are within an appropriate distance (<10 nm) of an upconverting nanoparticle (UCNP). The UCNPs absorb the long-wavelength light and emit light with higher energy to excite the vicinal PSs.^22^ Due to the requirement of a pulsed laser to trigger the TP-PDT, to combine TP-PDT and PTT in a single laser-activatable platform based on a PTA/PS hybrid, a pulsed laser-excitable PTA, which has a maximal absorption matched with the TP-absorption wavelength of the PS is required. Nevertheless, while there have been significant achievements in developing TP-PSs, only a few studies on dual TP-PDT/PTT combined therapy have been conducted.^23−25^

Plasmonic nanomaterials, especially gold NPs, have exhibited promising phototherapeutic potential, particularly in PTT and bioimaging, owing to their excellent biocompatibility, adjustable localized surface plasmon resonance (LSRP), and high photothermal performance.^26,27^ Interestingly, the integration of PSs and plasmonic metal could result in enhanced PDT due to the improved light absorption of the PSs when its absorption is in resonance with the LSRP of the plasmonic metal.^28−30^ Furthermore, the enhanced local electromagnetic field on the surface of plasmonic metal causes a significant enhancement in the nonlinear optical properties, such as TP-absorption cross-section and TPE-FL of the vicinal species.^31−34^ In particular, gold nanostars (AuNSs), thanks to their high TPAC and superb photothermal conversion efficiency, exhibit a promising potential to serve as PTA and TP-luminescence contrast agents under low-power pulsed laser irradiation.^35,36^ Moreover, compared to the well-known gold-based PTA, gold nanorods, which undergo shape deformation upon pulsed laser irradiation,^37^ AuNSs offer the possibility of developing low-power single-pulsed laser-triggered TP-PDT/PTT based on PTA/PS hybrids.^38^

**Scheme 1:**
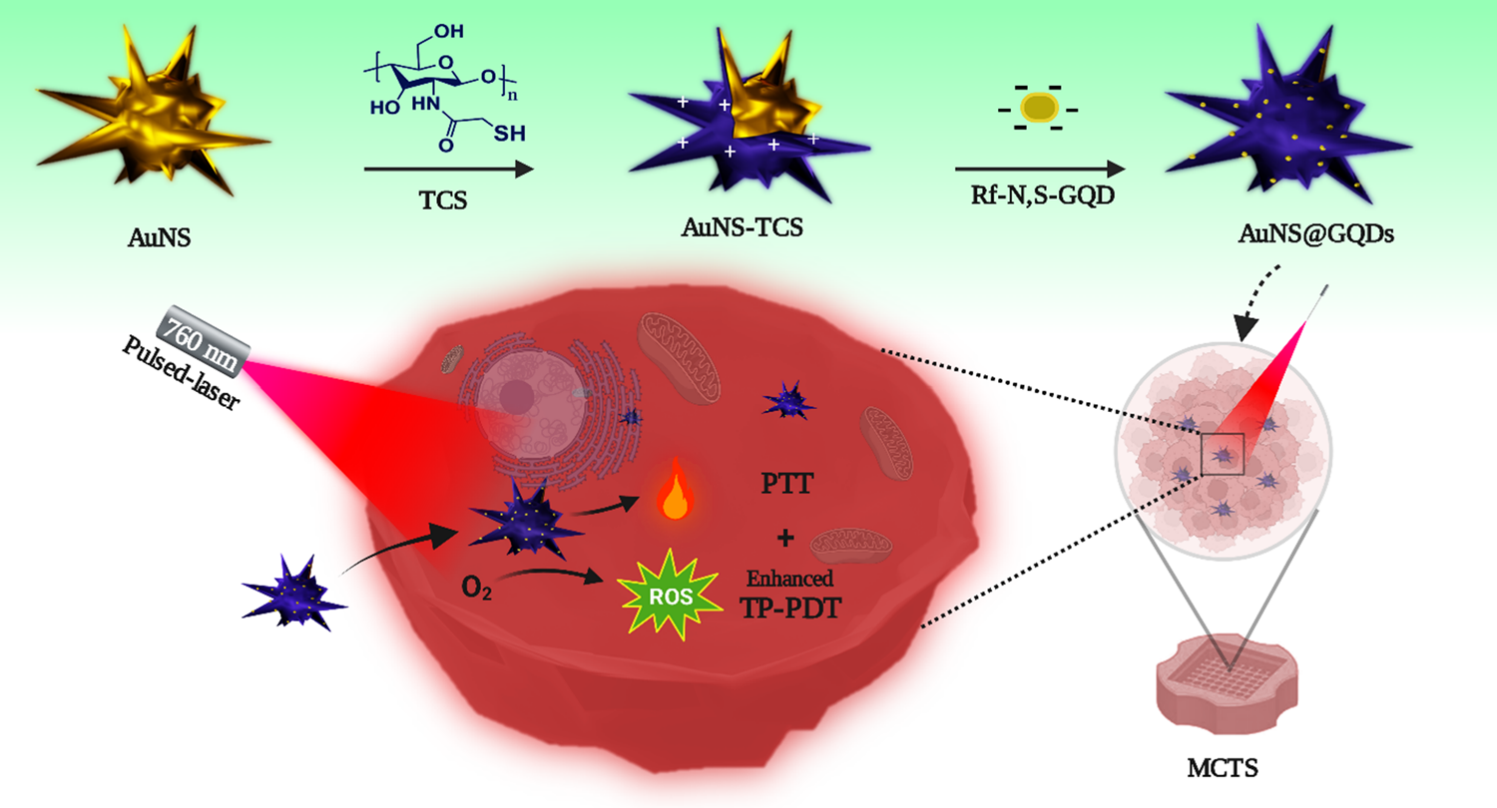
Construction Process of AuNS@GQDs and Their Phototherapeutic Processes in MCTSs

In our previous work, we synthesized a riboflavin-conjugated N-doped graphene quantum dot (Rf-N-GQD) and found it to be a promising tumor cell targeting TP-PS.^39^ In this study, we optimized the multistep synthesis procedure of Rf-N-GQD to a single-step pyrolysis method and doped sulfur into it to improve its phototherapeutic performance. Then, we designed and constructed a nanohybrid based on electrostatic adsorption of the negatively charged Rf-N,S-GQDs onto the surface of positively charged thiolated chitosan-coated AuNS (AuNS-TCS) to combine TP-PDT and PTT using a single platform (Scheme 1). We finely tuned the LSRP of AuNS to optimize its overlap with the TP-absorption wavelength of Rf-N,S-GQD and attain maximum synergistic therapeutic effects under single laser irradiation. Interestingly, upon low-power pulsed laser irradiation (200 mW·cm^–2^, lower than maximum permissible exposure threshold of skin (0.4 W.cm^–2^ at 850 nm)), we achieved an enhanced ^1^O_2_ generation alongside promising photothermal performance.^40^ 2D and 3D cellular model analyses revealed significantly superior TP-PDT/PTT therapeutic outcomes compared to individual PDT or PTT. As a result, this study demonstrates the promising potential of low-power single-pulsed laser-triggered TP-PDT/PTT using the plasmonic metal/semiconductor nanohybrid and highlights the synergistic therapeutic effects resulting from the combined PDT/PTT and plasmon-enhanced TP-absorption phenomenon, which leads to enhanced TP-PDT.^15,41,42^

## 2 Materials and Methods

### 2.1 Materials

3-mercaptopropionic acid (3-MPA), chloroauric acid (HAuCl4·3H2O), riboflavin (Rf), indocyanine green (ICG), anhydrous citric acid (CA), and ethylenediamine (EDA) were purchased from Thermo Fisher Scientific. Ascorbic acid (AA), chitosan (low molecular weight), thioglycolic acid, and silver nitrate (AgNO_3_) were supplied from Sigma-Aldrich. Thiolated chitosan (TCS) was synthesized according to a previously reported method.^43^ All reagents and solvents were utilized without further purification. Milli-Q water (DI-water, 18.2 MΩ·cm^–1^) was employed for the preparation of the all-aqueous solution.

### 2.2 Instruments

A Kratos AXIS Ultra HAS spectrometer was employed to perform the X-ray photoelectron spectra (XPS) analysis. A Lambda 35 (Perkin Elmer) UV–vis spectrophotometer and Synergy MX multimode microplate reader (Bio Tek) were employed to record the UV–vis and FL spectra, respectively. A Perkin Elmer Frontier Fourier transform infrared (FTIR) was used to obtain the FTIR spectra. The TEM images were obtained after drying of 5 μL nanoparticles (NPs) suspension on a cooper grid (300 mesh) followed by analyzing using a Zeiss-EM10C system. The HR-TEM images and the selected-area electron diffraction (SAED) patterns were obtained by an FEI Tecnai G^2^ F20 TEM brochure device. A Bruker 400 MHz was considered to obtain ^1^H NMR spectra. Hydrodynamic sizes and zeta-potentials were measured by Nano ZS90 (Brookhaven Inst. Corp.).

### 2.3 Synthesis of Rf-N,S-GQDs

One-pot thermal pyrolysis was carried out to synthesize the Rf-N,S-GQDs using CA, EDA, and 3-MPA as carbon, nitrogen, and sulfur sources, respectively. Initially, 1.9 g CA, 1 mL EDA, and 200 μL of 3-MPA were mixed in a 20 mL beaker and heated at 200 °C for 10 min. Then, 400 mg of Rf was added, followed by 5 min further heating at 200 °C. Subsequently, the dark-brown product was dropwise added to 50 mL of 10 mg·mL^–1^ NaOH under vigorous stirring overnight. After filtering the large particles using a 0.22 μm syringe filter and neutralization by 1 M HCl, the resultant dispersion was dialyzed (1000 Da MWCO) against Milli-Q water for 2 days to remove the unreacted reagents. Finally, the dialyzed dispersion was stored at 4 °C. UV–vis spectroscopy was utilized to quantify the released Rf from the dialysis bag to determine the Rf conjugation efficiency. As a control, N,S-GQD was also prepared using the same procedure without Rf addition.

### 2.4 Synthesis of AuNS

AuNS with an LSRP band centered at 750 nm was prepared through a well-known seed-mediated growth procedure with a slight modification.^44^ The seed NPs were prepared according to the Turkevich method.^45^ Briefly, 5 mL of citrate solution (1 wt %) was injected into a boiling solution of 95 mL of 0.5 mM HAuCl_4_, while it was being vigorously stirred. The citrate-capped gold NPs (GNPs) with diameter of 13 ± 2 nm were obtained after 15 min. AuNS were prepared by adding 200 μL of the previously prepared seed dispersion to a 20 mL vial containing 10 mL of 0.25 mM HAuCl_4_ and 10 μL of 1 M HCl while being gently stirred. After 1 min, 50 μL of 6 mM AgNO_3_ and 50 μL of 100 mM AA were injected with a 5 s delay, respectively. Formation of a bluish-green dispersion immediately after the injection of AA indicated the synthesis of AuNSs was successful.

### 2.5 Synthesis of AuNS-TCS

A prepared solution of TCS (consisting of 1 mL of solution in Milli-Q water, with a concentration of 5 mg·mL^–1^, and pH = 5) was slowly injected dropwise to a 5 mL volume of as-prepared AuNS dispersion. After moderate stirring for 4 h, the AuNS-TCS core–shell was washed three times through centrifugation (20 min, 2000 *g*) and redispersion in 5 mL Milli-Q water to remove the unadsorbed TCS.

### 2.6 Synthesis of AuNS@GQDs

Regarding the surface charges of the Rf-N,S-GQD (−15 mV) and AuNS-TCS (+34 mV), electrostatic adsorption was used to anchor the Rf-N,S-GQDs onto the surface of AuNS-TCS. Rf-N,S-GQD (100 μL, stock solution), and 5 mL of as-prepared AuNS-TCS were mixed under moderate stirring overnight. Then, centrifugation (2000*g*, 20 min) was employed to remove free Rf-N,S-GQDs. UV–vis was considered to quantify unadsorbed Rf-N,S-GQDs to determine the adsorption content.

### 2.7 Measurement of Extracellular ^1^O_2_ Generation

^1^O_2_ was detected using ICG as a selective probe.^46^ A mixture of 3.8 mL of ICG (25 μg·mL^–1^) and 200 μL of AuNS@GQDs (2 mg·mL^–1^) in the presence or absence of the NaN_3_ (^1^O_2_ scavenger, 200 μM) was irradiated using a light-emitting diode (LED) light (365 nm, 3 mW·cm^–2^). At different exposure times, the UV–vis spectrum of the under-irradiation dispersion was recorded. Furthermore, the same procedure was applied for equivalent concentrations of the Rf-N,S-GQD, and AuNS-TCS to evaluate the combinatorial effect on the ^1^O_2_ generation. ICG solution (25 μg·mL^–1^) also was illuminated under the same condition as the control.

### 2.8 Measurement of TPAC of the Rf-N,S-GQD

TPAC was determined by comparing the TPE-FL of Rf-N,S-GQD under different excitation wavelengths with rhodamine B as a reference.^47,48^ A Ti-Sapphire (680–840 nm, 80 MHz, and 90 fs) laser was employed to irradiate the samples. All experimental conditions such as detection and excitation were adjusted with negligible reabsorption to avoid its effect on the results. The TPAC at each excitation wavelength was calculated using the following equation:
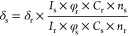
δ = TPAC, *I* = integrated fluorescence intensity, φ = photoluminescence quantum yield, *C* = concentration, and *n* = refractive index. s and r represent sample and reference, respectively.

### 2.9 Photothermal Performance Evaluation

The AuNS@GQDs (100 μg·mL^–1^), AuNS-TCS (80 μg·mL^–1^), and Rf-N,S-GQD (20 μg·mL^–1^) dispersion in phosphate-buffered saline (PBS, pH: 7.4) were subjected to NIR laser irradiation (Ti-Sapphire, 760 nm, 80 MHz and, 90 fs) for 5 min, using different power densities (200 or 400 mW·cm^–2^). The temperature was measured at each timepoint using a thermal infrared camera (Testo 865). The pH of the PBS buffer was 7.4.

### 2.10 Cells and Culturing

Caco-2 clone (a human colorectal adenocarcinoma cell line), human foreskin fibroblasts (HFF-1), and MCF-7 (a human breast cancer cell line) were supplied by American Type Culture Collection (ATCC, USA). Caco-2 clone and HFF-1 cells were cultured in Dulbecco’s modified eagle’s medium high glucose (DMEM) (Biowest, France), supplemented with 10% (v/v) fetal bovine serum (FBS) (Biochrom), 100 IU·mL^–1^ penicillin, and 100 μg·mL^–1^ streptomycin (Biowest, France) plus 1% (v/v) nonessential amino acids 100× (Gibco, USA). The MCF-7 cell line was maintained in DMEM/F12 (Gibco, USA) supplemented with 10% (v/v) FBS and 100 IU·mL^–1^ penicillin and 100 μg·mL^–1^ streptomycin. All cells were maintained in tissue culture flasks at 37 °C, 5% CO_2_, and in a water-saturated atmosphere within an incubator.

### 2.11 Cellular Cytotoxicity Assay

MCF-7 (10^4^ cells/well), Caco-2 clone (2 × 10^4^ cells/well), and HFF-1 (5 × 10^3^ cells/well) were seeded in 96-well plates using a complete medium and were maintained in the incubator for 24 h. The next day, the medium was discarded, and the cells were washed thrice with PBS 1×, followed by the incubation with 200 μL of the NPs dispersion in the respective complete medium to each well, at concentrations ranging from 0 to 100 μg·mL^–1^, for either 4 h or 24 h incubation time, at 37 °C, and 5% CO_2_. It also tested a negative control, a positive control, and a blank, corresponding to cells treated with Triton X-100 of 1% (v/v), cells incubated with just complete medium, and medium without cells, respectively. After each incubation time, the medium was discarded, and cells were washed thrice with PBS 1× to discard the samples. Each well then received 100 μL of fresh medium, followed by 20 min irradiation using LED light (365 nm and 3 mW·cm^–2^). Next, another 4 h incubation was done in an incubator. Subsequently, the cells were rinsed three times utilizing PBS 1×. Then, 200 μL of 20% (v/v) resazurin in the medium was added to each well, and the cells were left to incubate for 2 h. A microplate reader was employed to measure the FL intensity at λ_ex_ = 530 nm and λ_em_ = 590 nm. The same procedure, but without the irradiation step, was performed to evaluate the dark toxicity of the NPs. Thus, after either 4 or 24 h incubation with the NPs, 20% (v/v) resazurin in the medium was added and the FL intensity was assessed after 2 h of incubation. Each condition was replicated six times, and the resulting data were normalized based on the positive control with 100% metabolic activity.

### 2.12 Uptake Analysis

MCF-7 and HFF-1 were seeded in the 35 mm glass-bottom confocal dishes (ibidi, Germany), at 4 × 10^5^ cells/well and 1 × 10^5^ cells/well, respectively. The following day, cells were rinsed thrice with PBS 1× and treated with AuNS@GQDs in DMEM (2 mL, 100 μg·mL^–1^) for 2 h and 4 h. After treatment, cells were washed thrice with PBS 1× and fixed with 2% (v/v) PFA in PBS for 20 min at room temperature (RT), followed by permeabilization with 0.25% (v/v) TritonX-100 in PBS (500 μL) at RT for 10 min. Subsequently, cells were blocked with 10% (v/v) FBS in PBS (1 mL, 30 min at RT) and labeled with Alexa Fluor 546 phalloidin (1:100, 1 mL, 20 min) for actin staining. After three washes with PBS, nuclei were stained with DAPI (500 ng.mL^–1^, 20 min). Lastly, a confocal laser scanning microscope (CLSM) (Leica SP5, Germany) was employed to capture FL images by detecting Rf FL (490–550 nm) as the NPs signal.

### 2.13 Intracellular ROS Detection

To detect intracellular ROS, 2′,7′-dichlorodihydrofluorescein diacetate (DCFH-DA) was utilized as a probe in living cells. MCF-7 cells were seeded in a 35 mm confocal dish (ibidi, Germany) at 4 × 10^5^ cells/well for 24 h. Then, the cells were incubated with AuNS@GQDs in DMEM (2 mL, 100 μg·mL^–1^) for 4 h. After three washes with PBS, DCFH-DA (20 μM) was added to the cells and incubated for 30 min. Next, TPE (Ti-Sapphire, 760 nm, 80 MHz, 90 fs, and 200 mW·cm^–2^) was employed to trigger ROS generation. Finally, 4% (v/v) PFA in PBS was utilized to fix the cells and, then, the FL of the 2′,7′-dichlorodihydrofluorescein (DCF) (490–550 nm) was observed using the CLSM.

### 2.14 Live/Dead Cell Staining

MCF-7 cells were seeded (4 × 10^5^ cells/well) in confocal dishes (ibidi) 24 h prior to the experiment. Next, the cells were incubated with AuNS@GQDs in DMEM (2 mL, 100 μg·mL^–1^) for 4 h. After washing the cells thrice with PBS, phototherapeutic induction was executed using TPE (Ti-Sapphire, 760 nm, 80 MHz, 90 fs, and 200 mW·cm^–2^). Staining of live and dead cells was achieved utilizing Calcein-AM (2 μM, 500–550 nm) and propidium iodide (PI, 10 μM, 600–640 nm), respectively. Finally, FL imaging was conducted using a CLSM.

### 2.15 MCF-7 Multicellular Tumor Spheroids (MCTSs) Preparation

A previously established model was followed to prepare MCTSs of MCF-7 cells.^49^ Briefly, preheated agarose solution [2% w/v in NaCl (0.9%, w/v)] was cast into 3D micromolds (3D Petri Dish, from MicroTissues Inc.) with 81 circular recesses and left to solidify. The molds were then incubated with a complete medium for at least 2 h in a 12-well plate. Then, a suspension of MCF-7 cells (190 μL, 2.13 × 10^6^ cells/mL, approx. 5000 cells/MCTS) was added to the mold and left to settle for 30 min. Next, 2 mL of medium was carefully added to each well, and the MCTSs were incubated at 37 °C and 5% CO_2_ atmosphere, with medium change every other day.

### 2.16 MCTS Size Measurement

MCTS size and morphology were analyzed through Brightfield microscopy (ZOE Fluorescent Cell Imager, Bio-Rad Laboratories). The measurements were carried out by measuring two diameters per MCTS, and at least nine MCTSs were analyzed per mold using ImageJ software to calculate the average MCTS size on different days.

### 2.17 NP Biocompatibility and Phototoxicity Study in MCF-7 MCTSs

The relative viability of the MCF-7 MCTSs was determined by adenosine triphosphate (ATP) quantification, which is proportional to the viable cell counts.^50^ Initially, MCTSs were prepared as described above, and after 7 days of culture, they were collected and seeded in 96-well plates with three spheroids in each well, containing 100 μL of complete medium. Subsequently, 100 μL of the NPs dispersion in medium (6.25, 12.5, 25, 50, 100, and 200 μg·mL^–1^) were added to each well, and the MCTSs were incubated under the same incubation time and irradiation procedure as the 2D model. To measure the ATP content, 100 μL of CellTiter-Glo 3D Reagent (Promega Corporation, USA) replaced 100 μL of the medium in each well at the desired timepoint. The plates were shaken for 5 min (100 rpm), followed by 25 min incubation at RT, and then, luminescence was recorded using a multimode microplate reader.

To analyze the TP-toxicity of AuNS@GQDs, the MCTSs were incubated with the nanohybrid at a concentration of 100 μg·mL^–1^ for 24 h in the incubator. Then, the TP-irradiation (Ti-Sapphire, 760 nm, 80 MHz, 90 fs, and 200 mW·cm^–2^) was applied for 5 min to trigger the TP-PDT/PTT. After 4 h of further incubation in the incubator, the ATP content was measured as described above. In order to evaluate the TP-PDT and PTT efficiency individually, the same procedure was performed with MCTSs incubated with either Rf-N,S-GQDs (20 μg·mL^–1^) or AuNS@GQDs (100 μg·mL^–1^, NaN_3_ 100 μM), respectively.

### 2.18 ROS Detection in the MCF-7 MCTSs

ROS generation in MCTSs was detected using DCFH-DA. After 7 days of culture, MCTSs were collected and transferred to a 35 mm confocal dish for incubation with either AuNS@GQDs (100 μg·mL^–1^) with or without NaN_3_ or Rf-N,S-GQDs (20 μg·mL^–1^) for 24 h. After removing the old medium and washing the MCTSs three times with PBS 1×, they were incubated with 20 μM of DCFH-DA for 1 h. Next, TP-irradiation (Ti-Sapphire, 760 nm, 80 MHz, 90 fs, and 200 mW·cm^–2^) was used to activate TP-PDT for 5 min, and the green FL of the DCF was detected using the CLSM.

### 2.19 Statistical Analysis

The data are expressed as mean ± SD. Either the student’s *t* test (two-tailed) or one-way ANOVA was applied to calculate the significant differences between two groups and multiple groups, respectively. It is worth noting that the statistical significance was described as *P* < 0.05 (*), *P* < 0.01 (**), and *P* < 0.001 (***).

## 3 Results and Discussions

### 3.1 Synthesis and Characterization of AuNS@GQDs

**Figure 1:**
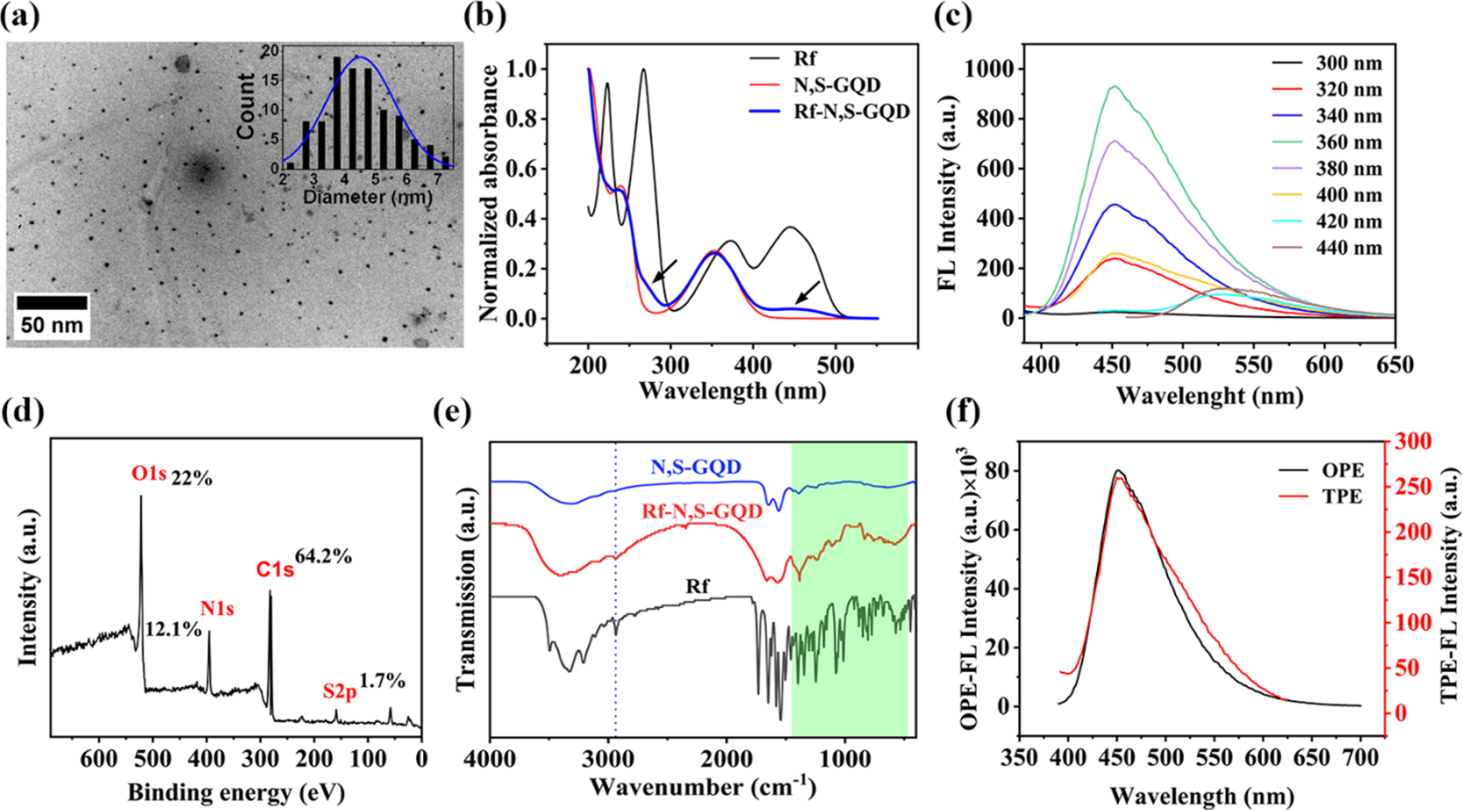
Characterization of Rf-N,S-GQD. (a) TEM image (inset: size distribution). (b) UV–vis spectra. (c) FL spectra of Rf-N,S-GQD. (d) XPS survey spectrum of Rf-N,S-GQD. (e) Comparing FT-IR spectra of N,S-GQD before and after Rf conjugation. (f) OP- and TPE-FL spectra of Rf-N,S-GQD (λ_OPE_ = 365 nm and λ_TPE_ = 760 nm).

Rf-N,S-GQDs were successfully synthesized by thermal pyrolysis of CA, EDA, and 3-MPA as carbon, nitrogen, and sulfur sources, respectively. Rf was conjugated to the surface and edges of N,S-GQDs at high temperatures by adding in the last stage of pyrolysis. The TEM image (Figure 1a) clearly shows a uniform size distribution with an average size of approximately 4.5 nm (Calculated using ImageJ analysis software by measuring the diameter of more than 100 NPs) (Figure 1a, inset). The optical properties were analyzed utilizing UV–vis absorbance and FL spectroscopy. In the UV–vis spectrum of Rf-N,S-GQD (Figure 1b), the maximum absorbances at 240 and 350 nm correspond to the π–π* transition of C=C of sp^2^ C domain and *n*–π* transitions of C=O, respectively. Furthermore, two characteristic absorption peaks similar to those of Rf at 280 and 445 nm confirmed the successful incorporation of Rf into the NPs. The emission spectra of Rf-N,S-GQD exhibited an inconspicuous shift under different excitation wavelengths due to its monodisperse size (Figure 1c),^51^ and an emission band centered at 520 nm upon excitation at 440 nm assigned to the conjugated Rf. The Rf content was determined to be 6% (w/w) by quantitative UV–vis analysis. The surface elements and composition of Rf-N,S-GQD were characterized using XPS, which resulted in the discovery of four significant peaks. These peaks were assigned to S 2p, C 1s, N 1s, and O 1s, with energy levels of 159, 282, 395, and 522 eV, respectively (Figure 1d). The analysis revealed that the composition of Rf-N,S-GQD consisted of 64.2% C, 12.1% N, 22% O, and 1.7% S. The existence of Rf in the Rf-N,S-GQD NPs was further confirmed by FTIR analysis (Figure 1e). To evaluate the TP property of Rf-N,S-GQD, its TPAC was determined at different excitation wavelengths from 680 to 840 nm. The maximum TPAC was measured to be as high as 33,000 Goeppert Mayer upon excitation at 760 nm, consistent with previous reports (Figure S1).^48,52^ As shown in Figure 1f, similarly to the OPE-FL, TPE-FL demonstrated a broad emission with a maximum intensity centered at 450 nm, indicating the successful TPE of Rf-N,S-GQD upon pulsed laser irradiation. Furthermore, the negligible change in the UV–vis absorbance spectrum of Rf-N,S-GQD upon pulsed laser irradiation reveals its acceptable photostability (Figure S2).

**Figure 2:**
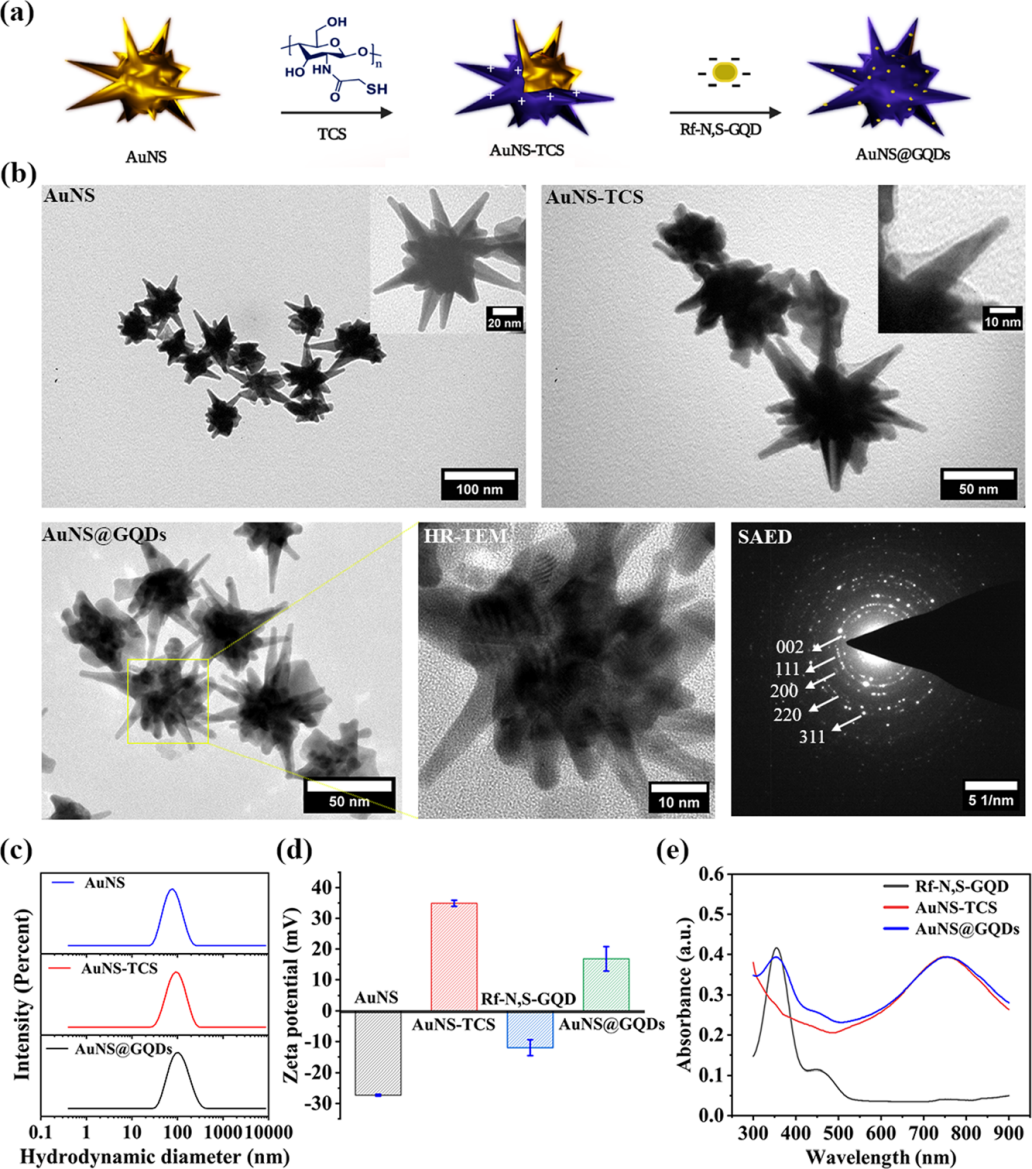
(a) Nanohybrid preparation scheme, (b) TEM and HR-TEM images, and SAED pattern, (c) hydrodynamic size distribution, and (d) zeta potential of AuNS, AuNS-TCS, and AuNS@GQDs. Data are presented as mean ± SD (*n* = 3). (e) UV–vis spectra.

The preparation steps of the nanohybrid are illustrated in Figure 2a. AuNSs were synthesized using a well-known seed-mediated and surfactant-free method.^44^ The procedure is based on the growing and branching of GNPs (13 ± 2 nm, Figure S3) in an acidic solution of Au^3+^ using AgNO_3_ and AA. By adapting the seed concentration, the LSRP of AuNS was adjusted around 750 nm, matched with the wavelength that Rf-N,S-GQD has maximum TPAC. The TEM images (Figure 2b) revealed that the pristine AuNSs have a uniform core size of 45 ± 2 nm and an average tip–tip size of around 70 nm, which is consistent with the determined hydrodynamic size using dynamic light scattering (DLS) analysis (z-average: 78 ± 1.3 nm) (Figure 2c).

Subsequently, TCS (Figure S4) was introduced to obtain AuNS-TCS core–shell NPs via electrostatic adsorption and Au–S bond formation. The positively charged layer provided by the TCS shell resulted in excellent colloidal stability (Figure S5), and electrostatic adsorption potential to adsorb negatively charged Rf-N,S-GQs. The TEM image showed a homogeneous layer of TCS on the surface of AuNS, and the zeta potential shifted drastically from −27.3 to +34.9 mV (Figure 2d), which indicates successful shell formation. Additionally, the hydrodynamic size slightly increased by around 11 nm after modification. The effective coating of TCS was further confirmed by FTIR spectroscopy, where the characteristic bands of the TCS appeared in the FTIR spectrum of the AuNS-TCS (Figure S6). The variations in the surrounding environment of AuNS after coating with TCS led to a slight red-shift of around 10 nm in its LSRP peak due to the higher refractive index of TCS compared to that of water (Figure S7).^53^

After successfully preparing AuNS-TCS, it was subsequently decorated with Rf-N,S-GQDs through electrostatic adsorption, resulting in small black dots on the surface of AuNS-TCS visible in the TEM and HR-TEM images. The extra crystal pattern (002) that emerged in the SAED pattern of AuNS@GQDs confirmed the crystal plane of Rf-N,S-GQDs, compared to AuNS (Figure S8). Additionally, the effective integration of Rf-N,S-GQD into the nanohybrid was confirmed by the appearance of its characteristic absorption peaks and FL emission in the AuNS@GQDs spectra after modification (Figures 2e and S9). The Rf-N,S-GQD content was determined (∼20% w/w) through UV–vis spectroscopy analysis of the unadsorbed NPs separated by centrifugation (Figure S10). On top of all that, the increment in hydrodynamic size and the reduction in the zeta potential of AuNS-TCS (from +34.9 to 14.6 mV) after Rf-N,S-GQD adsorption further confirmed the successful formation of AuNS@GQDs (Figure 2c,d).

### 3.2 ROS Generation Measurements of AuNS@GQDs upon OPE

**Figure 3:**
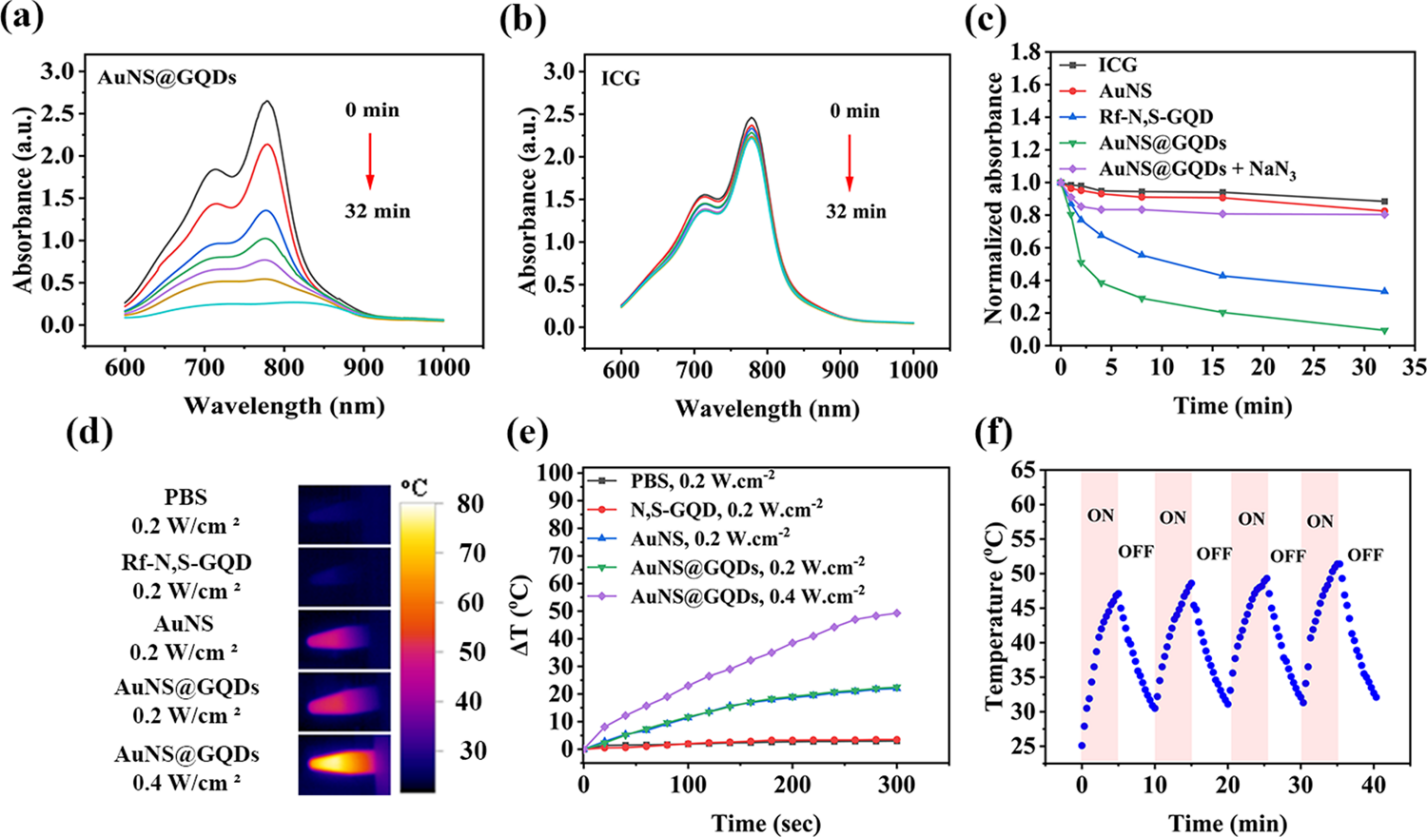
(a) UV–vis spectra of ICG in the presence of AuNS@GQDs (100 μg·mL^–1^) upon LED light (365 nm, 3 mW·cm^–2^) irradiation during 32 min. (b) UV–vis spectra of ICG upon LED light irradiation during 32 min. (c) Normalized absorbance of ICG at 780 nm in the presence of different NPs upon LED light irradiation. (d) Photothermal images and (e) temperature curves of different NPs (100 μg·mL^–1^) in PBS upon 5 min pulsed laser irradiation. (f) Raising/falling temperature curve of AuNS@GQDs dispersion (100 μg·mL^–1^) during four on/off laser cycles (200 mW·cm^–2^).

The PDT performance largely hinges on the ROS generation ability, particularly the ^1^O_2_, of PSs.^54^ To evaluate this capacity, AuNS@GQDs were irradiated with LED light (365 nm, 3 mW·cm^–2^) in an aqueous solution while monitoring the UV–vis absorbance spectra of ICG as a ^1^O_2_ selective probe.^46^ The presence of AuNS@GQDs under irradiation led to a significant decrease in the maximum absorbance of ICG (780 nm), indicating the efficient ^1^O_2_ generation ability of the nanohybrid (Figure 3a). In contrast, in the absence of the nanohybrid, the absorbance spectra of ICG exhibited only minor changes under irradiation (Figure 3b). The addition of NaN_3_, a ^1^O_2_ scavenger, resulted in no significant change in the absorption spectra of ICG during illumination, indicating the predominance of type-II PDT, in which ^1^O_2_ is the mainly generated ROS by the PS (Figure S11). Moreover, to investigate the plasmonic effect of AuNS on the ^1^O_2_ generation ability of Rf-N,S-GQD in the nanohybrid, the ^1^O_2_ production rates of AuNS and Rf-N,S-GQDs were individually examined under the same conditions (Figures 3c, S12, and S13). Compared to the individual Rf-N,S-GQDs, a substantial increase in the ^1^O_2_ production rate of Rf-N,S-GQDs in the nanohybrid was observed. This enhancement can be attributed to the plasmon-enhanced ^1^O_2_ generation effect, as previously reported in the literature.^28,29^

### 3.3 Photothermal Performance of AuNS@GQDs

The photothermal activity of AuNS@GQDs was assessed extracellularly using pulsed laser irradiation (Ti-Sapphire, 760 nm, 80 MHz, 90 fs, 200 mW·cm^–2^, and 5 min), and real-time temperature was monitored using a thermal IR camera. As shown in Figures 3d and S14, the temperature of the nanohybrid aqueous dispersion (100 μg·mL^–1^) rose to 48.6 °C and 73.2 °C after 5 min irradiation at 200 and 400 mW·cm^–2^, respectively. Meanwhile, negligible temperature changes (∼2 °C) were observed for PBS or Rf-N,S-GQD solution (Figure 3e). The as-prepared AuNS dispersion also demonstrated a similar temperature profile as AuNS@GQDs, indicating that AuNS could retain its photothermal performance in the nanohybrid. Moreover, photothermal stability was evaluated by recording the temperature changes during four successive laser on/off cycles, which showed negligible changes and demonstrated admirable photothermal stability (Figure 3f). Therefore, a laser power of 200 mW·cm^–2^ was selected for in vitro phototherapeutic studies, following the American National Standard Institute guidelines, to avoid the risks associated with high-power lasers.^40^

### 3.4 (Photo-)cytotoxicity and Cellular Uptake Analysis

**Figure 4:**
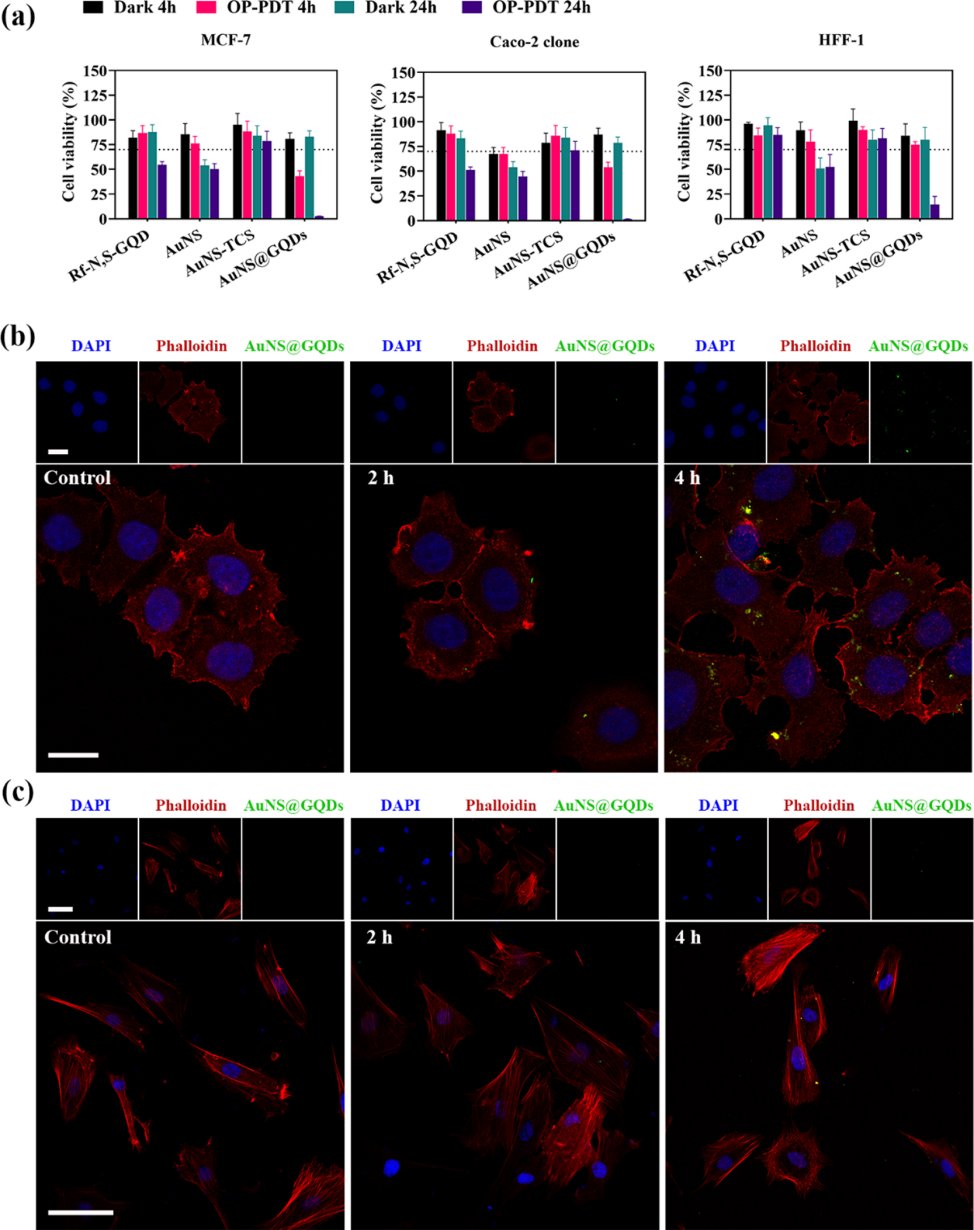
(a) Relative cell viability of the MCF-7, Caco-2 clone, and HFF-1 incubated with the AuNS@GQDs (100 μg·mL^–1^), AuNS (80 μg·mL^–1^), AuNS-TCS (80 μg·mL^–1^), and Rf-N,S-GQD (20 μg·mL^–1^) upon mentioned conditions. (b) and (c) CLSM images of the MCF-7 and HFF-1 cells incubated with AuNS@GQDs (100 μg·mL^–1^), respectively. The scale bars represent 20 μm and 100 μm for (b) and (c), respectively.

The (photo-)cytotoxicity of AuNS, AuNS-TCS, Rf-N,S-GQDs, and AuNS@GQDs, in MCF-7, Caco-2, and HFF-1 were investigated by a resazurin-based assay. Cells were incubated with various concentrations (0–100 μg·mL^–1^) of NPs for 4 and 24 h. All NPs showed high cell viability (>70%) after 24 h incubation in the dark condition (Figure 4a). Under irradiation, a reduction in cell viability was noticed, which was dependent on both the dose of NPs and time of incubation (Figures S15–S17). AuNS@GQDs exhibited higher phototoxicity compared to Rf-N,S-GQDs, which may be related to the plasmon-enhanced ^1^O_2_ generation effect, as shown in Figure 3c, demonstrating the efficient intracellular OP-PDT potential of the nanohybrid.

Subsequently, the cellular internalization of AuNS@GQDs (100 μg·mL^–1^) was studied in MCF-7 and nontumor HFF-1 cell lines using CLSM to detect the Rf emission as NP signal.^55,56^ The green FL of the nanohybrid in MCF-7 cells increased with the incubation time, and a cytoplasmic accumulation was observed using co-staining with DAPI and phalloidin (Figure 4b). Moreover, a lower FL intensity was observed in HFF-1 cells compared to MCF-7 cells after 4 h, which may be due to the high level of riboflavin transporters family (RFVTs) on the surface of MCF-7 cells (Figure 4c). Therefore, the presence of Rf moieties on the surface of AuNS@GQDs may enhance its cellular uptake in cancer cells with overexpressed RFVTs on their surface, as reported in previous studies.^57^

### 3.5 Intracellular ROS Generation upon TP-Irradiation

**Figure 5:**
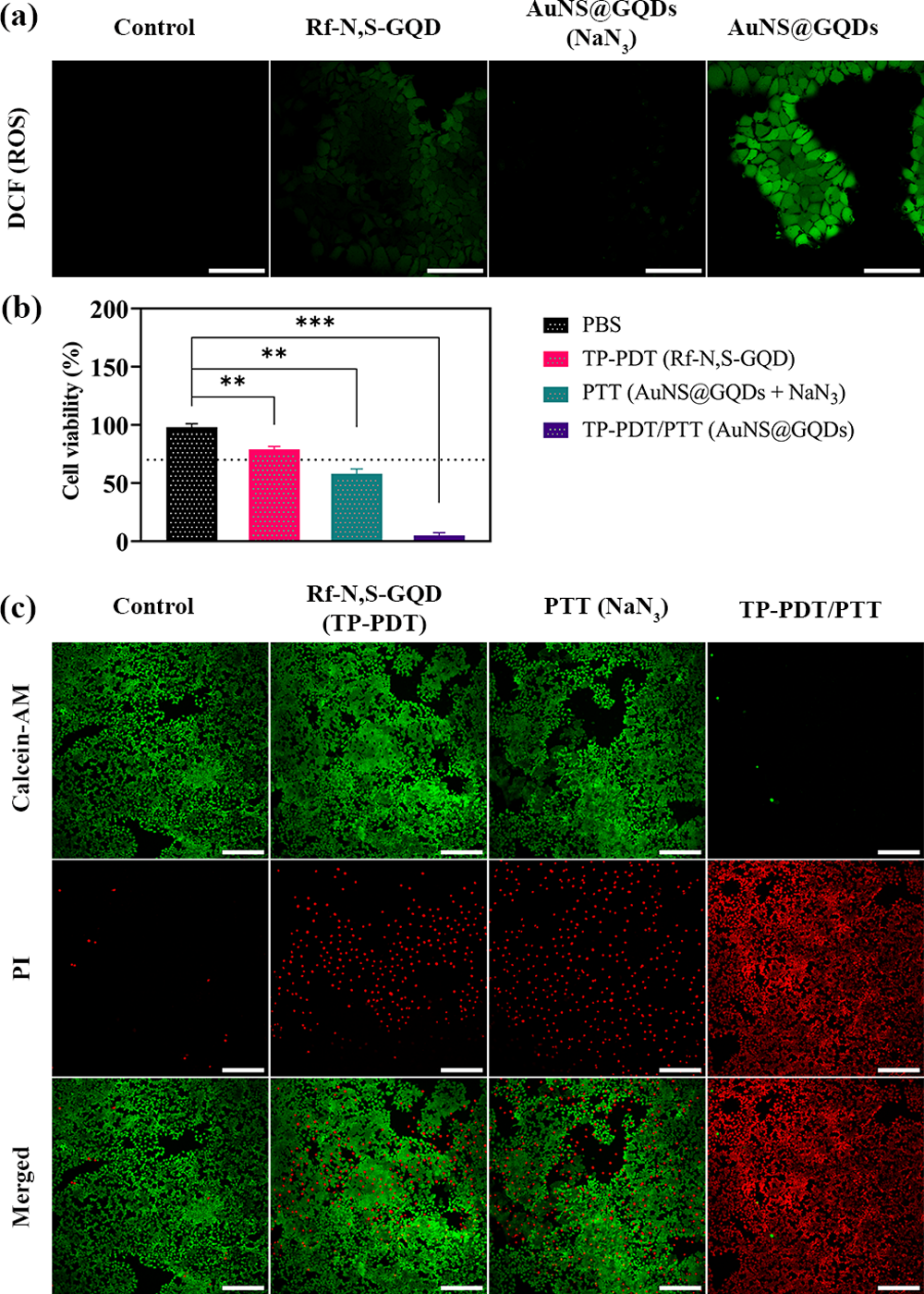
(a) CLSM images of the MCF-7 cells co-incubated with the NPs and DCFH-DA (20 μM) after pulsed laser irradiation. The scale bars represent 100 μm. (b) Cell viability of the MCF-7 cells treated with the AuNS@GQDs (100 μg·mL^–1^) in the presence and absence of NaN_3_(100 μM), and Rf-N,S-GQD (20 μg·mL^–1^) upon pulsed laser irradiation. (c) CLSM images of the MCF-7 cells co-incubated with the NPs and Calcein-Am/PI (2 μM/10 μM) upon pulsed laser irradiation. The scale bars represent 300 μM. The data are expressed as mean ± SD (*n* = 3). *P* < 0.01 (**) and *P* < 0.001 (***).

To assess the effectiveness of the nanohybrid in TP-PDT, intracellular ROS generation upon TP-irradiation was evaluated. MCF-7 cells were treated with AuNS@GQDs (100 μg·mL^–1^, 4 h), and DCFH-DA was used to quantify ROS production after irradiation with a pulsed laser (Ti-Sapphire, 760 nm, 5 min, 80 MHz, 90 fs, and 200 mW·cm^–2^). A bright green FL appeared in the cells incubated with AuNS@GQDs, revealing the efficient intracellular ROS generation upon TPE (Figure 5a). In contrast, the FL intensity in cells treated with Rf-N,S-GQDs (equivalent concentration) was weaker than in those treated with AuNS@GQDs, indicating the enhanced ROS generation ability of the nanohybrid. This can be attributed to the plasmonic effect of AuNS, which led to the TPAC enhancement of Rf-N,S-GQDs.^28,32^ Furthermore, the noticeable reduction in FL intensity after co-incubation with nanohybrid and NaN_3_ (100 μM) further confirmed ^1^O_2_ as the mainly generated ROS and type-II PDT pathway.

### 3.6 TP-PDT/PTT Combination Therapy

The exceptional photothermal effect and excellent intracellular ^1^O_2_ generation ability of AuNS@GQDs prompted us to explore its potential in TP-PDT/PTT combination therapy toward MCF-7 cells. Upon pulsed laser irradiation (Ti-Sapphire, 760 nm, 5 min, 80 MHz, 90 fs, and 200 mW·cm^–2^), the cell viability decreased to 4% in the cells treated with the AuNS@RGQDs (100 μg·mL^–1^, 4 h), indicating a high phototoxicity result (Figure 5b). To investigate the individual TP-PDT and PTT phototoxicity, cells were treated with Rf-N,S-GQD (equivalent concentration), and co-incubated with AuNS@GQDs and NaN_3_ (which limits the ^1^O_2_ effect), respectively. The phototoxicity from either PDT or PTT was lower compared to that of the combinatorial therapy, demonstrating a significant synergistic effect (TP-PDT/PTT > TP-PDT + PTT) due to the enhanced ^1^O_2_ generation capability of the nanohybrid. Calcein-AM (green) and PI (red) co-staining were employed to visualize cell death efficiency. Compared to the individual strategies, combinatorial therapy showed significant red FL, consistent with resazurin assay-based findings (Figure 5c). As a control, MCF-7 cells were irradiated without NPs incubation, demonstrating negligible PI-stained cells and suggesting that this low-power pulsed laser irradiation did not compromise the cells.

### 3.7 (Photo-)cytotoxicity Evaluation on the MCF-7 MCTS

**Figure 6:**
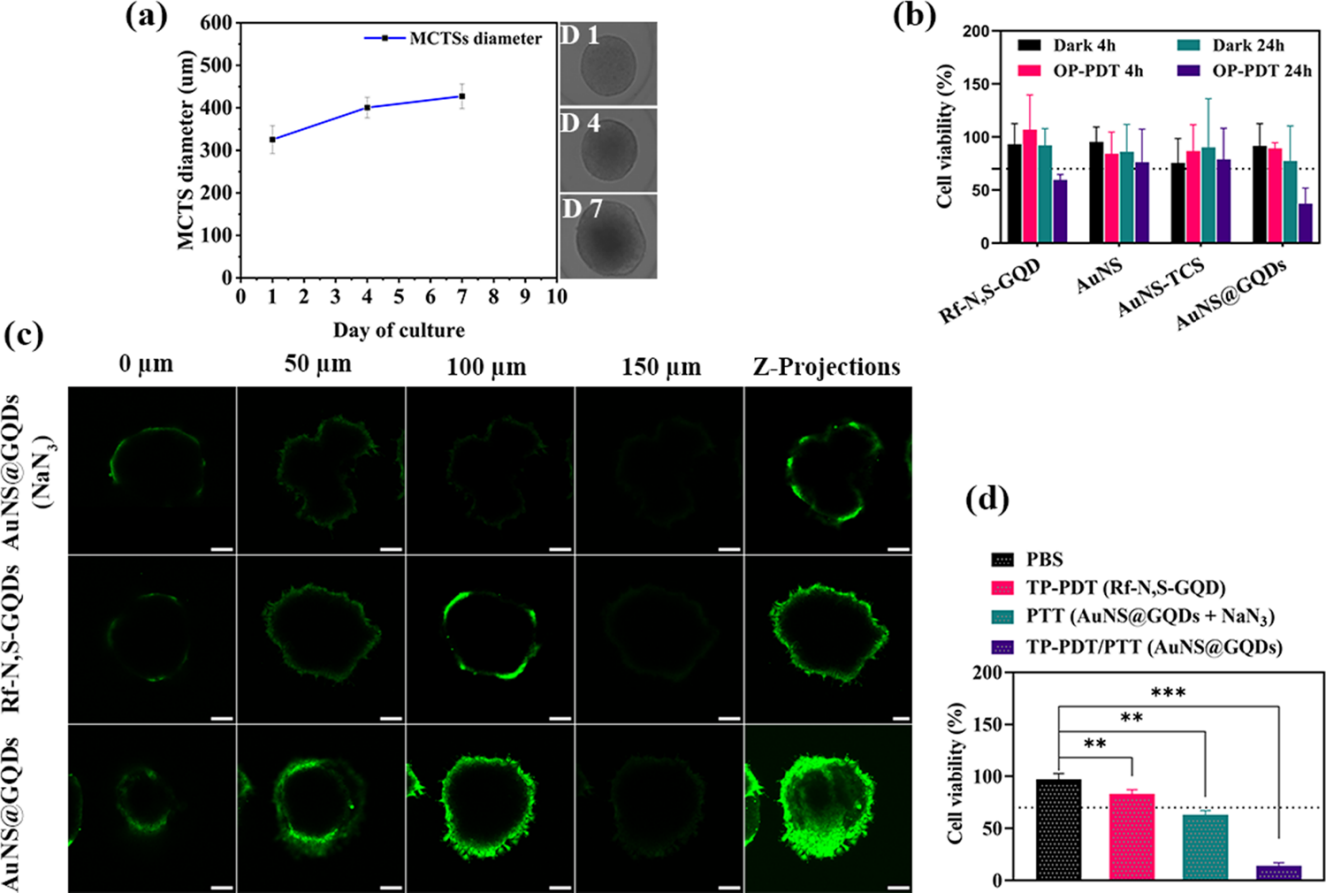
(a) Size of the MCTS during 7 days of culture. (b) (Photo-)cytotoxicity assay of AuNS@GQDs (100 μg·mL^–1^), AuNS (100 μg·mL^–1^), AuNS-TCS (100 μg·mL^–1^), and Rf-N,S-GQD (20 μg·mL^–1^) on the MCTSs in the dark and upon LED light (365 nm, 3 mW·cm^–2^, and 20 min). (c) CLSM images of the MCTSs co-incubated with the NPs (24 h) and DCFH-DA (20 μM, 1 h) under NIR irradiation. (d) Cell viability of the MCTSs incubated with the AuNS@GQDs (100 μg·mL^–1^) in the presence and absence of NaN_3_ (100 μM), and Rf-N,S-GQD (20 μg·mL^–1^) after NIR irradiation. The data are expressed as mean ± SD (*n* = 3). *P* < 0.01 (**) and *P* < 0.001 (***).

After evaluating the (OP- and TP-)PDT therapeutic efficiency of AuNS@GQDs in 2D monolayer cells, we investigated the (photo-)cytotoxicity and intracellular ROS generation ability of the nanohybrid toward 3D MCF-7 MCTSs. MCTSs are heterogeneous cellular aggregates that better mimic the in vivo model than 2D monolayer cells and can validate the results obtained from the monolayer models before exploring drug effects in animal models. The MCF-7 MCTS was prepared and reached a diameter of 450 nm after 7 days of culture before being incubated with the NPs (Figure 6a). The OP-PDT and dark toxicity of the NPs were evaluated under the same conditions as those for the 2D monolayer cells. All NPs demonstrated a high safety profile in the dark after 24 h of incubation (Figure 6b). Similar to the 2D monolayer model, a time- and dose-dependent phototoxicity was observed in the Rf-N,S-GQDs and AuNS@GQDs incubated group upon OP-irradiation (365 nm, 3 mW·cm^–2^, and 20 min) due to their ROS generation ability under UV irradiation (Figure S18).

To further study the TP-PDT therapeutic effect in MCF-7 MCTS, intracellular ROS generation in the NPs-treated MCTSs upon TP-irradiation (760 nm, 200 mW·cm^–2^, and 5 min) was visualized using the ROS probe DCFH-DA. Figure 6c shows a more pronounced green FL of DCF in the nanohybrid-treated MCTSs compared to the Rf-N,S-GQD treated MCTSs. Similar to the observed results in the 2D model, AuNS enhanced the ROS generation ability of the vicinal Rf-N,S-GQDs upon TP excitation due to the plasmon-enhanced ROS production effect. Furthermore, the MCTSs were co-incubated with the nanohybrid and NaN_3_ to evaluate the type of generated ROS upon pulsed laser irradiation. As observed in the 2D model, the negligible green FL in the presence of NaN_3_ revealed a type-II PDT pathway in which ^1^O_2_ is the mainly generated ROS.

The combined TP-PDT/PTT effect was further investigated on the 3D MCTSs. The MCTSs were incubated with NPs for 24 h after 7 days of culture, followed by TP-irradiation (760 nm, 200 mW·cm^–2^, and 5 min). The results showed that the cell viability significantly decreased to 14% under combination therapy, indicating efficient phototoxicity compared to individual TP-PDT or PTT (Figure 6d). Overall, these observations demonstrate the potential of AuNS@GQDs to compromise 3D cell architectures under TP-irradiation via synergistic TP-PDT/PTT combination therapy.

## 4 Conclusions

In summary, a novel nanohybrid using Rf-N,S-GQDs and AuNS-TCS as TP-excitable PSs and low-power pulsed laser-activatable PTA, respectively, was successfully developed. The nanohybrid exhibited simultaneous TP-PDT and PTT under single laser irradiation (200 mW·cm^–2^, 760 nm, 80 MHz, and 90 fs) owing to the spectral overlap between the TPE-wavelength of Rf-N,S-GQD and the LSRP of plasmonic AuNS. The study found an improved ^1^O_2_ generation ability upon OP- and TP-irradiation in both 2D monolayer cells and 3D MCTSs as a result of the plasmonic effect of AuNS. Moreover, the photothermal performance of AuNS was well-maintained in the nanohybrid, and an effective temperature increment was observed extracellularly under pulsed laser irradiation (200 mW·cm^–2^, 760 nm) for 5 min. Notably, the combination of TP-PDT/PTT in this system achieved a synergistic therapeutic effect and higher phototoxicity compared to individual TP-PDT or PTT. These findings suggest that AuNS@GQDs hold great promise in treating solid tumors and offer potential for in vivo analysis and clinical translation in future research.

## Supporting Information Available

Detailed characterizations of AuNS, TCS, Rf-N,S-GQD, AuNS@GQDs, e.g., TPACS, DLS, and ^1^H NMR spectra, ^1^O_2_ generation of the different species, SAED pattern, colloidal stability, photostability analysis, full cell-viability graphs of the different cell lines, and UV–vis absorbance and FL spectra (PDF)
