# Supplementary material for: Exploring Low-Power Single-Pulsed Laser-Triggered Two-Photon Photodynamic/Photothermal Combination Therapy Using a Gold Nanostar/Graphene Quantum Dot Nanohybrid
Source: ACS Appl Mater Interfaces. 2023 Apr 21;15(17):20811–21. doi: 10.1021/acsami.3c03578 (PMC10165604; doi:10.1021/acsami.3c03578)
Supplement: Supplementary file 1 — am3c03578_si_001.pdf [file am3c03578_si_001.pdf]

Supporting information

Exploring Low-Power Single Pulsed Laser-Triggered Two-Photon Photodynamic/Photothermal Combination Therapy Using a Gold Nanostar/Graphene Quantum Dot Nanohybrid

Amir Soleimany [a, b, c], *Sepideh Khoee* [c, *], *Sofia Dias* [a, b, d], *Bruno Sarmento* [a, b, e, *]

[a] i3S, Instituto de Investigação e Inovação em Saúde, Universidade do Porto, Rua Alfredo Allen 208, 4200-135 Porto, Portugal.

[b] INEB, Instituto de Engenharia Biomédica, Universidade do Porto, Rua Alfredo Allen 208, 4200-135 Porto, Portugal.

[c] Polymer Laboratory, School of Chemistry, College of Science, University of Tehran, Tehran, 14155-6455, Iran.

[d] ICBAS - Instituto de Ciências Biomédicas Abel Salazar, Universidade do Porto, Rua Jorge de Viterbo Ferreira 228, 4050-313 Porto, Portugal

[e] IUCS-CESPU, Rua Central de Gandra 1317, 4585-116 Gandra, Portugal.

[*] **Corresponding Author:** Email: khoee@khayam.ut.ac.ir

[*] **Corresponding Author:** Email: bruno.sarmento@i3s.up.pt

Table of contents:

Figure S1 TPAC of the Rf-N,S-GQD ………………………………………………… S3

Figure S2 Photostability analysis of Rf-N,S-GQD upon ……………………………... S3

Figure S3 Size distribution of the Gold nanoparticles …………………………………... S4

Figure S4 ^{1}H-NMR spectra of the chitosan and thiolated-chitosan ……………………... S4

Figure S5 Colloidal stability of the AuNS and AuNS-TCS ……………………………... S5

Figure S6 FTIR spectra of the AuNS and AuNS-TCS ………………………………….. S5

Figure S7 UV-vis spectra of the AuNS and AuNS-TCS …………………….………….. S6

Figure S8 SAED pattern of the AuNS ……………………………………...………… S6

Figure S9 FL spectra ……………………………………………………………….. S7

Figure S10 UV-vis absorption spectra of the AuNS-@GQDs... S7

Figure S11 UV-vis spectra of the ICG ………………………………………………… S8

Figure S12 UV-vis spectra of the ICG ……………………………………………….…… S8

Figure S13 UV-vis spectra of the ICG ………………………………………………… S9

Figure S14 The In-vitro IR thermal images ………………………………………… S9

Figure S15 Cell viability of MCF-7 cells …………………………………………...… S10

Figure S16 Cell viability of Caco-2 clone cells …………………………………..….. S11

Figure S17 Cell viability of HFF-1 cells …………………………………………..……. S12

Figure S18 Cell viability of MCF-7 MCTS ………………………………...…… S13

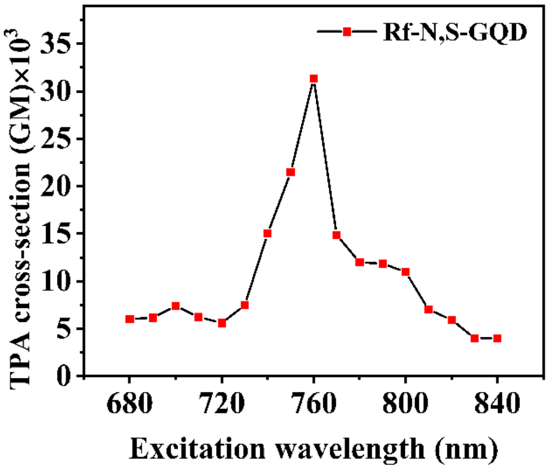

Figure S1. TPAC of the Rf-N,S-GQD(λ_{TPE} = 680-840 nm).

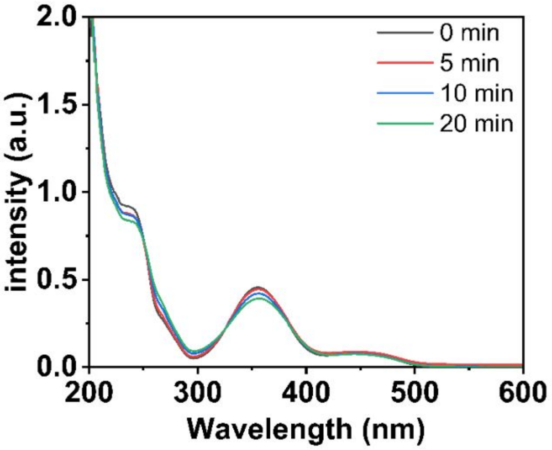

Figure S2. Photostability analysis of Rf-N,S-GQD upon pulsed laser irradiation.

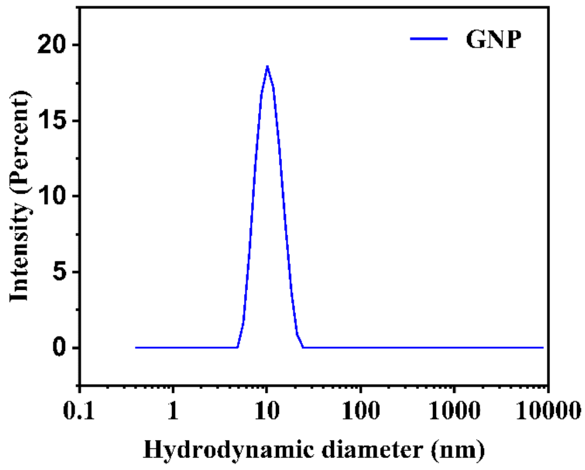

Figure S3. Size distribution of the Gold nanoparticles (GNPs, seed), recorded by DLS.

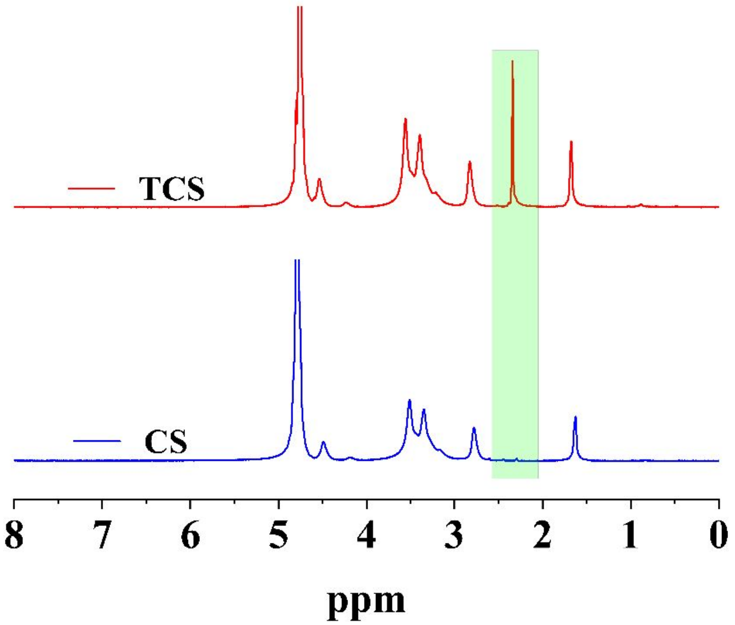

Figure S4. ^{1}H-NMR spectra of the chitosan and thiolated-chitosan (TCS).

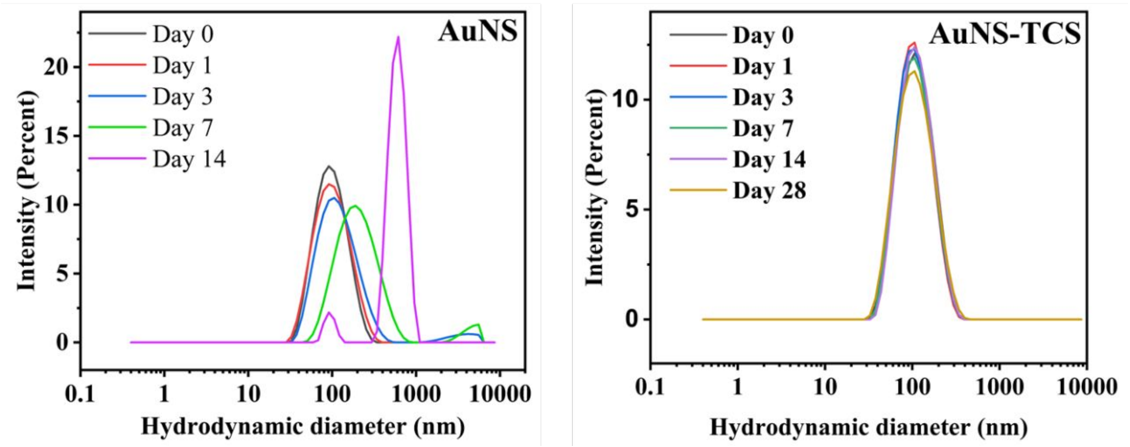

Figure S5. Colloidal stability of the AuNS and AuNS-TCS in PBS buffer (pH=7.4) at RT. Size distribution during 2 and 4 weeks, respectively.

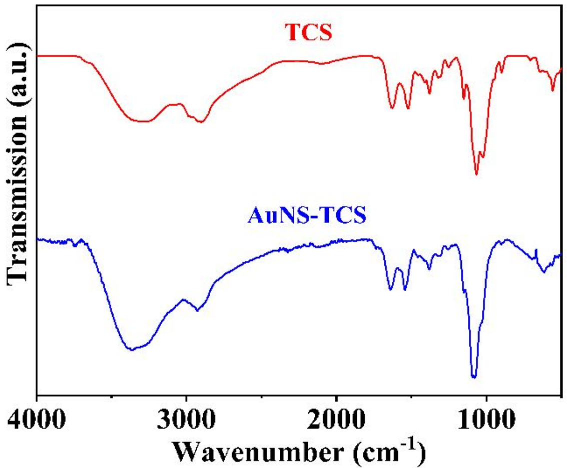

Figure S6. FTIR spectra of the AuNS and AuNS-TCS.

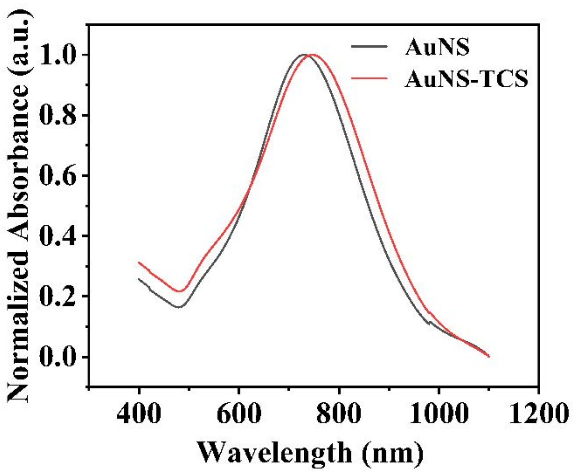

Figure S7. UV-vis spectra of the AuNS and AuNS-TCS.

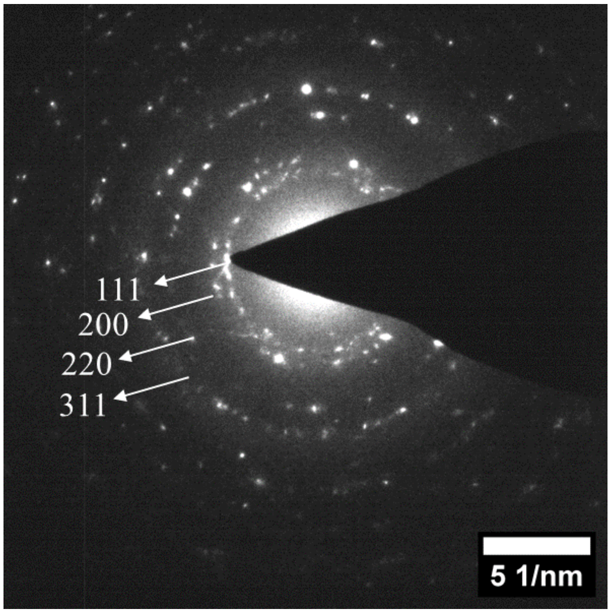

Figure S8. SAED pattern of the AuNS.

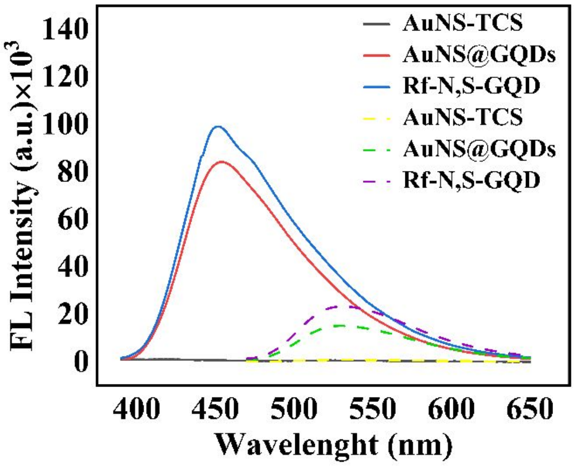

Figure S9. FL spectra (solid lines; λ_{ex} = 370 nm and dashed line; λ_{ex} = 450 nm).

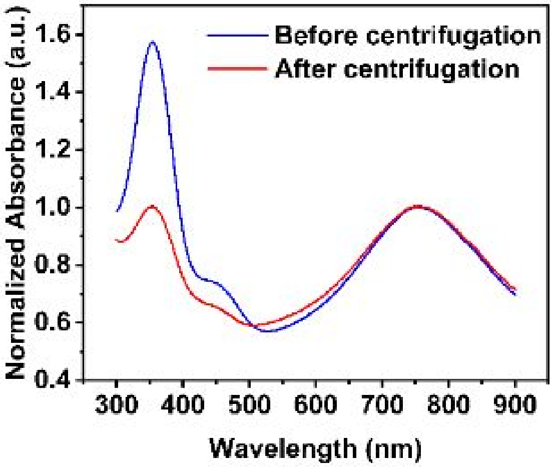

Figure S10. UV-vis absorption spectra of the AuNS-@GQDs before and after separation of the un-adsorbed Rf-N,S-GQDs by centrifugation.

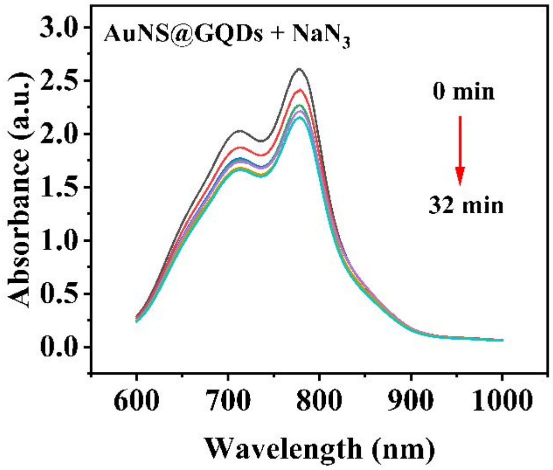

Figure S11. UV-vis spectra of the ICG upon LED light irradiation (365 nm, 3 mW.cm^{-2}).

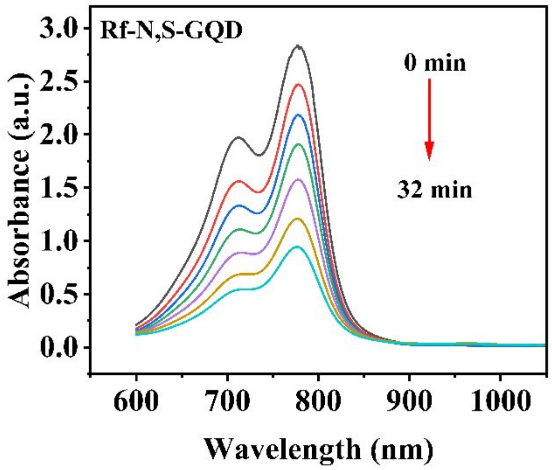

Figure S12. UV-vis spectra of the ICG upon LED light irradiation (365 nm, 3 mV.cm^{-2}).

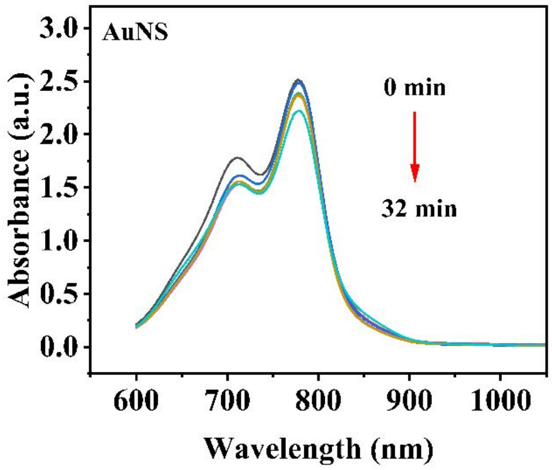

Figure S13. UV-vis spectra of the ICG upon LED light irradiation (365 nm, 3 mW.cm^{-2}).

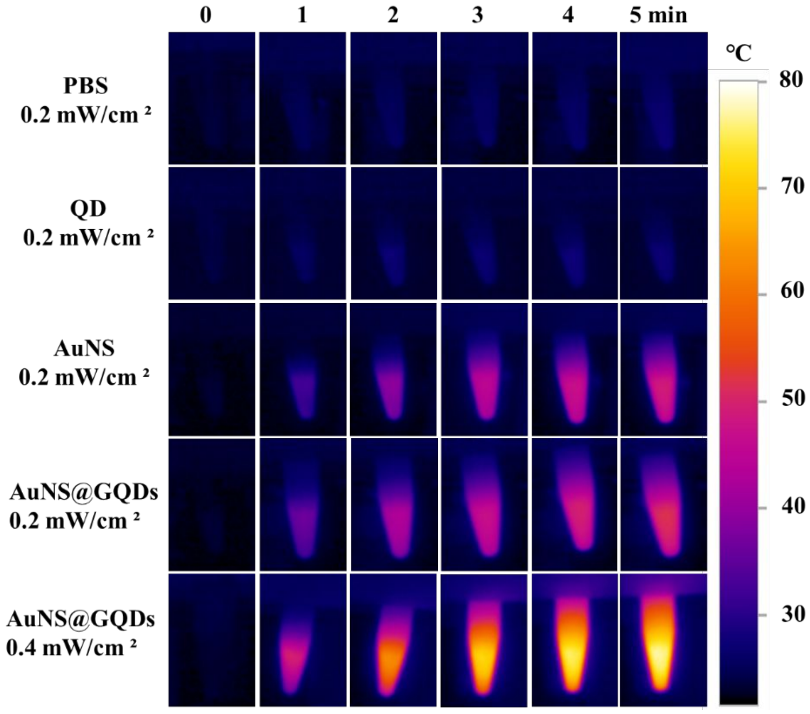

Figure S14. The In-vitro IR thermal images of different NPs as mentioned, during 5 min irradiation.

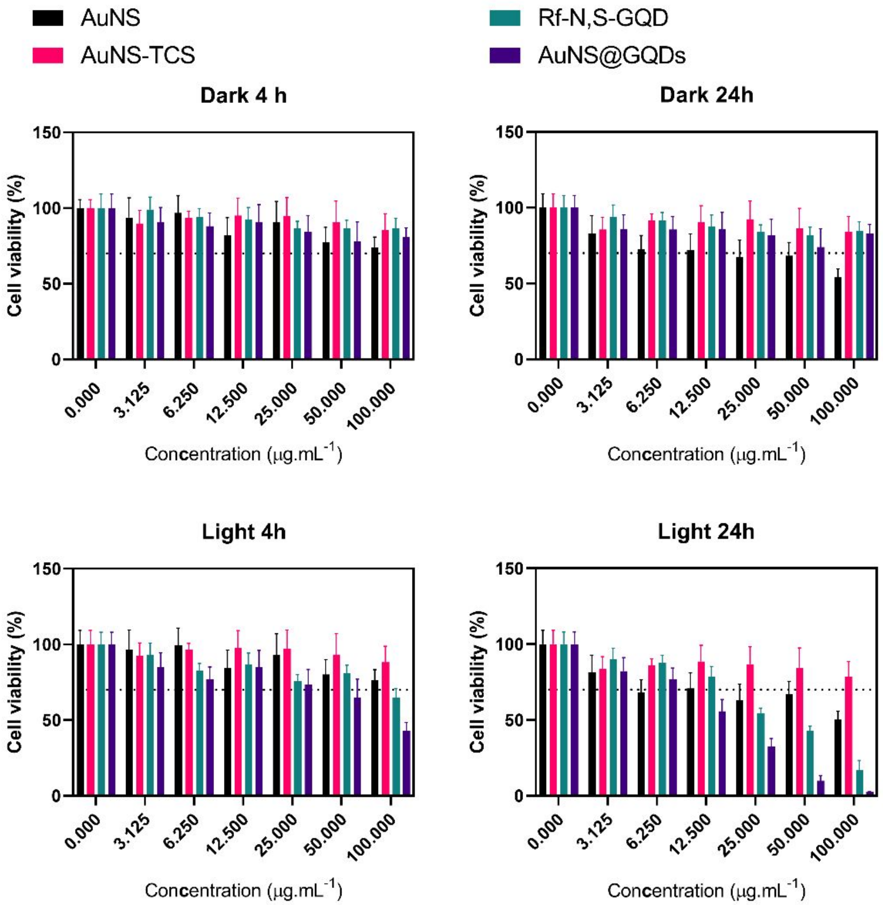

Figure S15. Cell viability of MCF-7 cells incubated with different NPs upon mentioned conditions. A LED light (365 nm, 3 mW.cm^{-2}, 20 min) was used to induce OP-PDT.

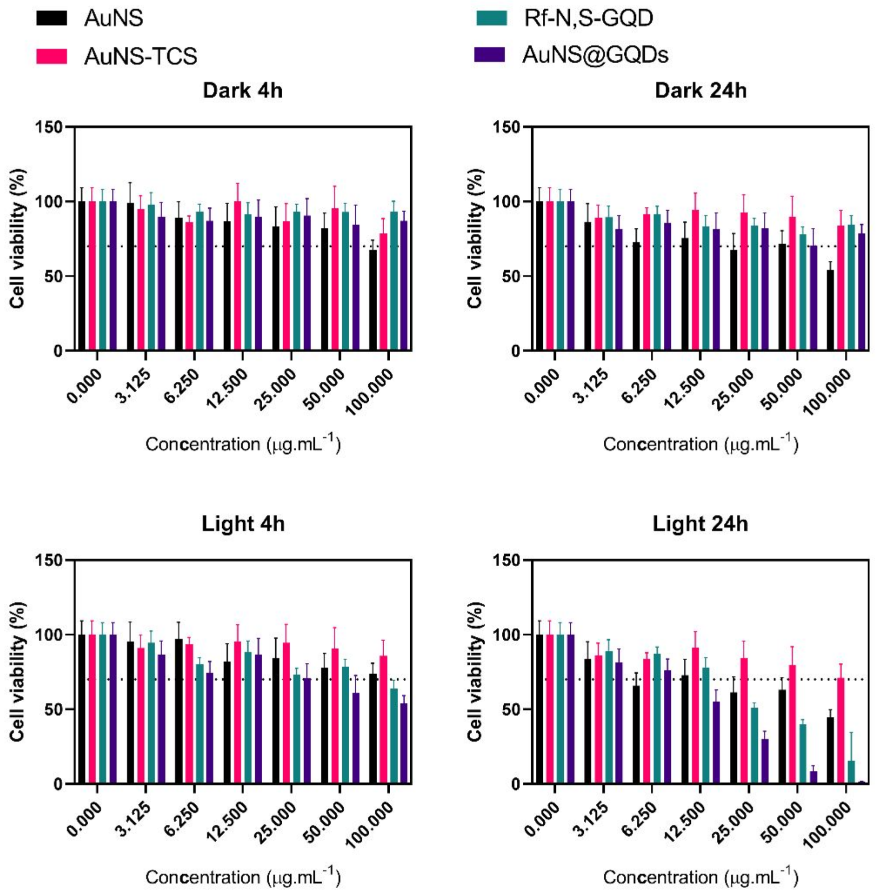

Figure S16. Cell viability of the Caco-2 clone cells incubated with different NPs upon mentioned conditions. A LED light (365 nm, 3 mW.cm^{-2}, 20 min) was used to induce OP-PDT.

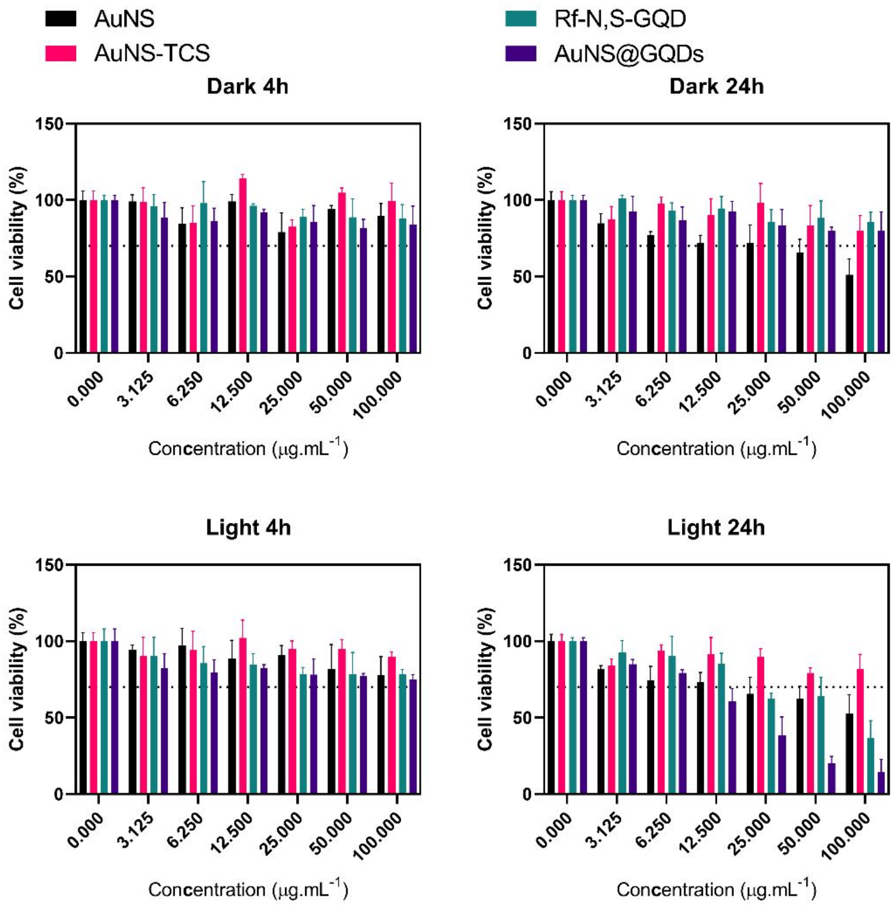

Figure S17. Cell viability of HFF-1 cells incubated with different NPs upon mentioned conditions. A LED light (365 nm, 3 mW.cm^{-2}, 20 min) was used to induce OP-PDT.

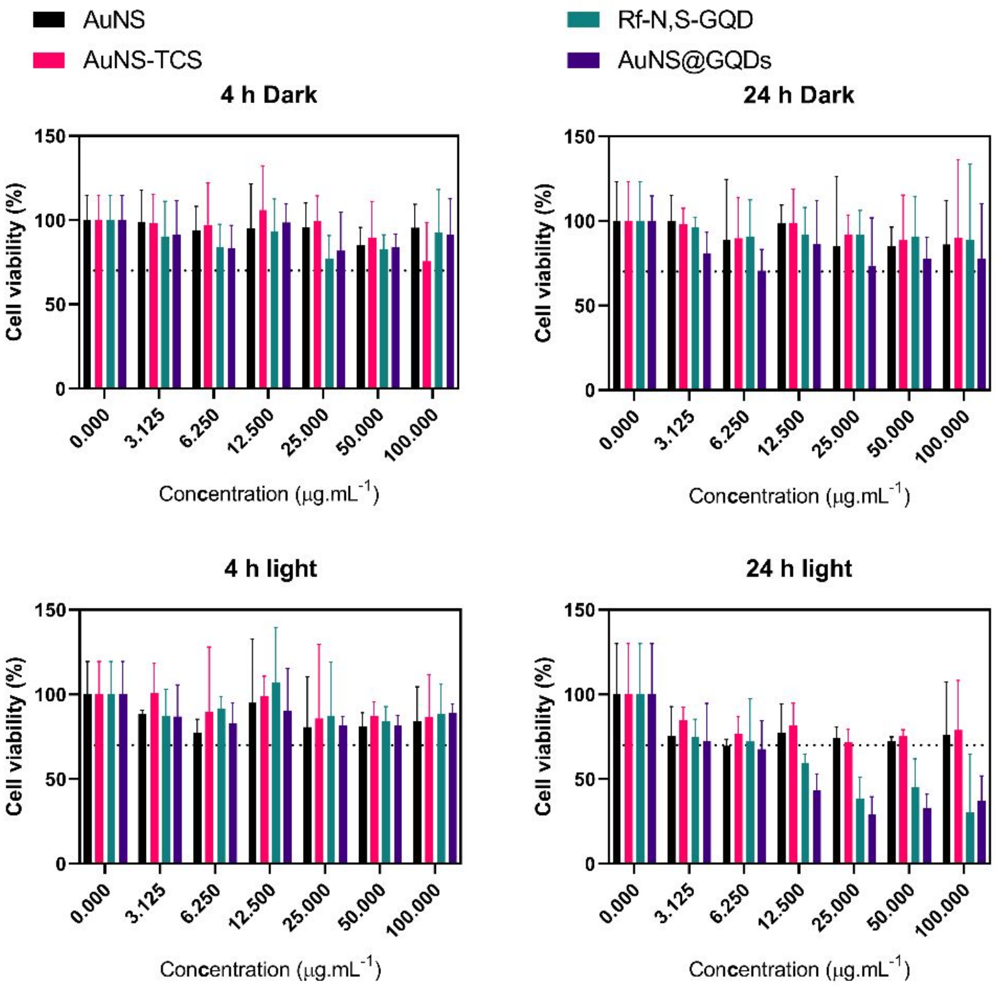

Figure S18. Cell viability of MCF-7 MCTS incubated with different NPs upon mentioned conditions. A LED light (365 nm, 3 mW.cm^{-2}, 20 min) was used to induce OP-PDT.
